# Reducing Stocking Densities and Using Cooling Systems for More Adapted Pigs to High Temperatures When Reared in Intensive Conditions

**DOI:** 10.3390/ani13152424

**Published:** 2023-07-27

**Authors:** Alexandra Contreras-Jodar, Damián Escribano, José Joaquin Cerón, Marina López-Arjona, Pau Aymerich, Carme Soldevila, Emma Fàbrega, Antoni Dalmau

**Affiliations:** 1 Animal Welfare Program, Institute of Agrifood Research and Technology (IRTA), 17121 Monells, Spain; alexandra.contreras@irta.cat (A.C.-J.); emma.fabrega@irta.cat (E.F.); 2 Interdisciplinary Laboratory of Clinical Analysis, Interlab-UMU, Regional Campus of International Excellence Campus Mare Nostrum, University of Murcia, 30100 Murcia, Spain; det20165@um.es (D.E.); jjceron@um.es (J.J.C.); 3 Department of Animal and Food Science, Universitat Autònoma de Barcelona, 08193 Bellaterra, Spain; marina.lopez.arjona@uab.cat; 4 Vall Companys Group, 25191 Lleida, Spain; paymerich@vallcompanys.es (P.A.); csoldevila@vallcompanys.es (C.S.)

**Keywords:** pigs, heat stress, mitigation strategies, cooling systems, stocking density, animal-based measures, welfare

## Abstract

**Simple Summary:**

Heat stress negatively affects animal welfare, health, and production efficiency, especially in pigs. Pigs have few functional sweat glands and are further hindered by a thick subcutaneous adipose tissue layer, which impedes heat dissipation in warm conditions. Outdoor pigs cope with hot periods by choosing their own environment (e.g., looking for shade and wallowing in mud). However, heat-stressed pigs raised in intensive, confined housing wallow in their manure to cool themselves, despite not being their preferred substrate, while avoiding contact with other pigs. In addition, pigs implement physiological adaptations in an attempt to cope with heat stress, with negative consequences on pigs’ performance and carcass quality, causing dramatic losses in the swine industry worldwide. For this reason, to counteract the impact of heat stress in intensive pig farming, the implementation of multiple mitigation strategies is required. Thus far, the effect of the use of cooling systems and/or reducing the stocking density on pig welfare and performance has not been quantified, and the results can be of interest to the swine industry.

**Abstract:**

This study is aimed at evaluating the effect of reducing stocking density and using cooling systems to mitigate the negative effects of high temperatures in growing pigs (females and castrated males) reared in intensive conditions (from 25 to 100 kg) during summer (June to October 2020). The experimental design was a 2 × 2 factorial where pigs were provided with an evaporative cooling system and/or raised at regular or at lower stocking densities (i.e., 0.68 to 0.80 m^2^/animal). Treatments were distributed in four different rooms containing sex-balanced pens with either castrated males or females. Temperature and humidity were recorded throughout the experiment, and the temperature–humidity index was calculated. Heat stress (HS) on pigs was measured through changes in animals’ performance, animal-based indicators (dirtiness and activity budget) and physiological indicators (neutrophil/lymphocyte ratio and hair cortisol). The use of cooling, lowering stocking density and the combination of both strategies had positive effects on pigs’ final body weight (+5 kg, +3 kg, +9 kg, respectively; *p* < 0.001). The prevalence of dirtiness was similar at the stocking densities tested, and no clear effect of the cooling system was found. Both mitigation strategies lowered the physiological indicators of stress, although only hair cortisone can be considered an indicator of HS. In conclusion, both mitigation strategies are effective in improving pig welfare and performance, especially when both are combined. The severity of the stocking density effect may depend on the severity of the temperature.

## 1. Introduction

Heat stress (HS) is a major environmental issue that jeopardizes animal welfare, health, and production efficiency in almost every livestock species [1,2]. Among domestic species, pigs are one of the most susceptible to HS since they have few functional sweat glands and are further hindered by a thick subcutaneous adipose tissue layer, which impedes sensible heat loss. Therefore, pigs rely on respiratory routes (i.e., panting) to dissipate heat [3], although it is not very efficient. Outdoor pigs cope with hot periods by choosing their environment (e.g., looking for shade, wallowing in mud and staying away from other pigs). However, heat-stressed pigs raised in intensive, confined housing wallow in their manure to cool themselves, despite not being their preferred substrate, while avoiding contact with other pigs [4]. However, at the stocking densities that pigs are usually reared in intensive conditions, they cannot avoid contact between individuals, especially at the fattening stage. If heat loss mechanisms are insufficient to maintain euthermia, pigs will implement various strategies to minimize heat production, such as decreasing physical activity, increasing water intake, and decreasing feed intake in order to reduce metabolic heat production [5]. While animals modify their behavior to cope with environmental conditions, their physiology is also modified to maintain euthermia. In fact, HS not only impairs animal welfare but also negatively affects pig performance and carcass quality [6,7], causing losses in the swine industry worldwide. For this reason, to counteract the impact of HS in intensive pig farming, the management of HS requires multiple mitigation strategies. These could range from physically modifying the environment using cooling methods [8] and reducing the stocking density, including feed additives and other dietary modifications [9,10]. Strategies related to physical modification of the environment are aimed at decreasing the air temperature or causing direct cooling to the pig’s skin by either the effect of water evaporation (misting, fogging, sprinkling, evaporative pads, drip cooling) or by the implementation of methods that enhance convection or heat exchange via conduction [8].

Combinations such as evaporation and convection using cooling and tunnel ventilation have synergistic effects, but they are known to have a high cost [11]. Effective, low-cost strategies include reducing stocking density, although no scientific literature is presumably available on pigs.

So far, the literature has been focused on the impact of HS and the effect of different mitigation strategies on pig physiology and productive performance but not on the outcomes of welfare indicators. Welfare indicators are based on the behavior and health of pigs and are aimed at collecting information concerning the stress response of the animal and its effects on it. For this, a series of measures must be used to evaluate the HS response from an animal welfare point of view. Along this line, the HS response can be measured using a combination of indicators such as observation of the animal (e.g., changes in behavior, clinical indicators), physiological indicators and changes in productive performance [12]. The aim of the present study was to evaluate the effect of cooling panels versus natural ventilation and reducing stocking densities on fattening pigs’ welfare under summer conditions with the hypothesis that either or both strategies will produce a positive effect on the coping capacities of pigs reared in hot climates.

## 2. Materials and Methods

### 2.1. Animals and Treatments

The study was carried out on a commercial farm of pigs located in Alcarràs, Lleida, Spain, from June to October 2020. According to the Köppen climate classification, this area has a cold semi-arid climate. Winters are humid and very cold, and summers are hot. The average annual precipitation is quite low, about 340 mm, with minimums in summer and winter and maximums in spring and autumn. It is not strange that, throughout the year, temperatures of a few degrees Celsius below zero can be registered in winter and up to more than 40 °C in summer. The summer of 2020 was especially hot, being the hottest in Spain since 1961. The commercial farm is formed of two buildings with a capacity of 1456 pigs each and divided into two rooms (4 rooms in total). Within each room, from 616 to 728 pigs were housed in a total of 56 pens. Each room is divided by a central corridor, one side containing only castrated males and the other side only females, that were never mixed. Males were castrated at 3 d old without anesthesia or analgesia on another commercial farm. The animals were a commercial cross of Lean Duroc × (Landrace × Large White). Animals entered the farm at two months old, weighing 25 kg. Once pigs in the first room achieved a mean weight of 100 kg, all pigs were sampled, and the study finished.

The treatments of the study consisted of providing an evaporative cooling system and reducing the stocking density from 13 pigs per pen (high density: 0.68 m^2^/animal) to 11 pigs per pen (low density: 0.80 m^2^/animal). The first density is based on the minimum space required by the EU regulation for pigs reaching 85 kg (0.65 m^2^ per pig), and the second considers a maximum of two pigs less per pen. Hence, the treatments were distributed as follows: a first room with a cooling system and 11 pigs per pen; a second room without a cooling system and 13 pigs per pen; a third room without a cooling system and 11 pigs per pen; and a fourth room, with a cooling system and 13 pigs per pen. The cooling system consisted of a mechanical evaporative cooling system. Evaporative cooling exploits the fact that water will absorb a relatively large amount of heat in order to evaporate. The temperature of dry air can be dropped significantly through the phase transition of liquid water to water vapor (evaporation). The cooling system in each room consisted of 3 fans Exafan EX50-1V (Zaragoza, Spain) with a cellulose panel, with a surface of 5.4 m^2^ and a thickness of 15 cm, with a speed air of 1.8 m per second and a flow rate of 32,000 m^2^/h (96,000 considering the three fans).

### 2.2. Thermal Conditions

Temperature and humidity were recorded throughout the experiment and used to calculate the temperature–humidity index (THI) according to the formula of the National Weather Service Central Region [13]. Temperatures and humidity were taken with two probes from the company Exafan (Zaragoza, Spain).

### 2.3. Animal-Based Indicators

Animals had a 15 d adaptation period, already placed at different stocking densities with all rooms naturally ventilated. Then, all animals were individually weighed, and the cooling treatments started. The trial lasted 81 d, when pigs were weighed, and average daily gain (ADG) was calculated on a pen basis. The dirtiness and activity budget were assessed at the end of the study, as described in the Welfare Quality Protocol for pigs [14]. On the one hand, dirtiness was assessed on individual animals in 10 balanced by sex (five females and five castrated males) pens per room, selected randomly at the beginning of the study (Room 1: *n* = 110 pigs; Room 2: *n* = 130; Room 3: *n* = 107; Room 4: *n* = 130). It consisted of scoring individual animals using a 3-point scale ranging from 0 to 2 according to the percentage of the pig’s body that was soiled with manure. A score of ‘0’ was given when it was less than 20%, ‘1’ when it was between 20% and 50%, and ‘2’ when it exceeded 50%. An animal scored with a ‘1′ was considered partially dirty and, consequently, considered a 0.5 dirty individual, while all the animals scored with a ‘2’ were considered a dirty animal.

On the other hand, the activity budget was assessed in animals of eight pens per room (Room 1: *n* = 86 pigs; Room 2: *n* = 107; Room 3: *n* = 83; Room 4: *n* = 105). The activity budget consisted of five scan samples made at 2.5 min intervals in each room, considering the following behaviors: ‘Negative social’, defined as aggressive behavior, including biting or any social behavior with a response from the disturbed animal; ‘Positive social’, defined as sniffing, nosing, licking and moving gently away from the animal without an aggressive or flight reaction from this individual; ‘Exploratory behavior’, defined as sniffing, nosing licking or chewing all features of the pen and play/investigation toward straw or other enrichment material, and ‘Resting’, when the animal is lying on the floor sleeping or just inactive. All of these definitions were taken from the Welfare Quality protocols for pigs [14].

### 2.4. Blood and Hair Samples

Blood and hair sampling was performed for quantification of biological markers of stress. For this, all animals from a total of four balanced by sex (two females and two castrated males) pens per room (16 in total) were sampled (Room 1: *n* = 44 pigs; Room 2: *n* = 52, Room 3: *n*= 44; Room 4: *n* = 52).

Blood samples were collected at midsummer, 10 d after a heat wave, for a complete hemogram to calculate the neutrophil/lymphocyte ratio as an indicator of mid-term stress [15].

Hair samples were collected individually at the end of the experiment by shaving an area of around 10 cm × 10 cm located on the pig’s dorsal rump, as described in Puppe et al. [16]. Cortisone was extracted as in Davenport et al. [17] and analyzed by means of AlphaLISA^®^, already validated for the hair of pigs [18]. Hair cortisone as a biological marker of chronic stress was quantified and expressed as pg of cortisone/mg of hair.

### 2.5. Statistical Analysis

SAS was used for the statistical analyses (SAS Institute Inc.; Cary, NC, USA). The measures, THI, neutrophil/lymphocyte ratio, cortisone, body weight (BW, initial and final) and ADG, were normally distributed and were analyzed by means of Proc Mixed. For the measure of final body weight, repeated measures were used with the pen as subject and initial body weight as covariable. For behavior indicators (resting, social and exploratory behavior) and dirtiness, a binomial distribution with Proc Genmod was used. In all cases, the fixed effects included were the presence or absence of cooling, males or females, stocking density (low or high) and all the interactions between factors. The least-square means of fixed effects (LSMEANS) were used for multiple comparisons. The differences were considered statistically significant when the *p*-value was lower than 0.05.

## 3. Results

### 3.1. Thermal Conditions

**Figure 1:**
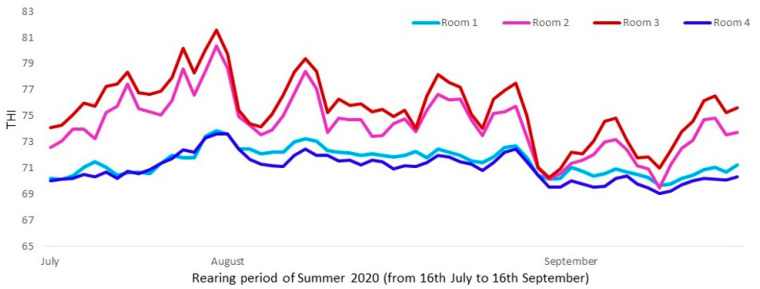
Mean daily temperature–humidity index (THI) in a pig farm according to the room during Mediterranean summer conditions. Room 1 was provided with cooling, and stocking density per pen was 0.80 m^2^/pig; Room 2 had no cooling, and stocking density per pen was 0.68 m^2^/pig; Room 3 had no cooling, and stocking density per pen was 0.80 m^2^/pig; Room 4 was provided with cooling, and stocking density per pen was 0.68 m^2^/pig.

The mean daily THI per room through the entire experimental period is shown in Figure 1. When statistically analyzed, rooms with cooling (rooms 1 and 4; THI = 71.3 ± 0.13) had lower (*p* < 0.001; F = 308.97; DF = 1/310) THI values in comparison to rooms without cooling (rooms 2 and 3; THI = 74.9 ± 0.13).

### 3.2. Animal-Based Indicators

Significant effects on initial BW were reported for cooling (*p* = 0.006; F =7.7; DF = 1/215), sex (*p* < 0.001; F = 17.4; DF = 1/215) and stocking density (*p* < 0.001; F = 27.01; DF = 1/215). Animals housed with a cooling system (35.8 kg ± 0.3 kg) weighed 1.0 kg more than animals housed without a cooling system (34.8 kg ± 0.3 kg). Males (36.3 kg ± 0.3 kg) weighed 2.1 kg more than females (34.3 kg ± 0.3 kg). Finally, animals housed in groups of 11 (36.4 kg ± 0.3 kg) weighed 2.3 kg more than pigs reared in groups of 13 (34.1 kg ± 0.34 kg). In addition, an interaction between cooling and stocking density was significant (*p* = 0.035; F = 4.5; DF = 1/215). In this case, animals with cooling systems in groups of 11 (37.5 kg ± 0.5 kg) weighed 3.4 kg more than pigs without a cooling system and in groups of 13 (34.1 kg ± 0.5 kg). Consequently, initial BW was used as a covariate in ADG and final BW models.

Final BW was affected by cooling (*p* < 0.001; F= 109.6; DF= 1/206), sex (*p* = 0.042; F = 4.2; DF = 1/206), stocking density (*p* < 0.001; F = 20.1; DF = 1/206) and the interaction between cooling × density (*p* = 0.046; F = 4.0; DF = 1/26). Animals housed with a cooling system (113.6 kg ± 0.3 kg) weighed more than animals housed without a cooling system (107.4 kg ± 0.3 kg). Males (112.4 kg ± 0.4 kg) weighed more than females (108.6 kg ± 0.3 kg), and animals housed in groups of 11 (113.1 kg ± 0.3 kg) weighed more than pigs reared in groups of 13 (107.7 kg ± 0.3 kg). In addition, animals raised with cooling systems in groups of 11 (118.4 kg ± 0.5 kg) weighed more than pigs without a cooling system and in groups of 13 (106.8 kg ± 0.4 kg). Finally, the initial BW, as covariable, was also statistically significant (*p* < 0.001; F= 499.33; DF= 1/206).

**Figure 2:**
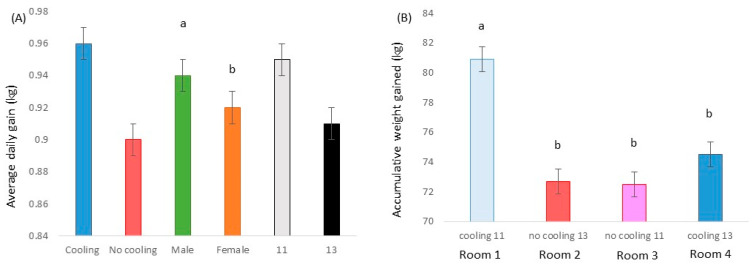
(**A**) Average daily gain (kg/day) for pigs housed with a cooling device or without, males or females and low or high stocking densities. Different letters mean significant differences at *p* < 0.05. (**B**) Accumulated body weight gain (kg) for animals in room with cooling system and low stocking density, room without a cooling system and high stocking density, room without a cooling system and low stocking density and room with a cooling system and high stocking density. Dirtiness was affected by sex (*p* < 0.001; χ^2^ = 52.1; DF = 1), males being dirtier than females, by a trend for density (*p* = 0.090; χ2 = 2.9; DF = 1) and by cooling × density interaction (*p* = 0.001; χ^2^ = 10.8; DF = 1) (Figure 3).

When ADG was considered, a cooling effect (*p* < 0.001; F = 18.3; DF = 1/164), a stocking density effect (*p* = 0.003; F = 9.1; DF = 1/164), a trend for sex (*p* = 0.073; F = 3.3; DF = 1/164) and cooling × stocking density interaction (*p* < 0.001; F = 12.6; DF = 1/164) were found. In the 81 days from the first time the animals were weighed (15 d after mixing), animals with a cooling system and 11 pigs per pen (89.9 kg) gained, in this period, 9.3 kg more than animals without a cooling system and 13 pigs (80.6 kg) per pen (Figure 2).

**Figure 3:**
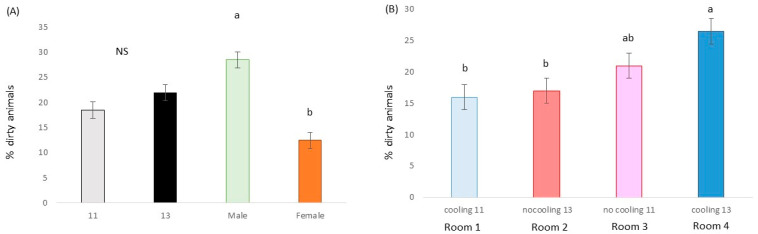
Percentage of dirty pigs according to: (**A**) the stocking density and sex, and (**B**) according to the experimental treatments (Room 1: with cooling system and 11 animals per pen; Room 2: no cooling and 13 animals per pen; Room 3: no cooling and 11 animals per pen; Room 4: with cooling and 13 animals per pen). Partially dirty pig (>20% but <50% of the body surface with manure) was computed as one-half pig. Dirty pig (>50% of the body surface with manure) was computed as one pig. Different letters mean significant differences at *p* < 0.05. NS means no significant differences at *p* > 0.05.

**Figure 4:**
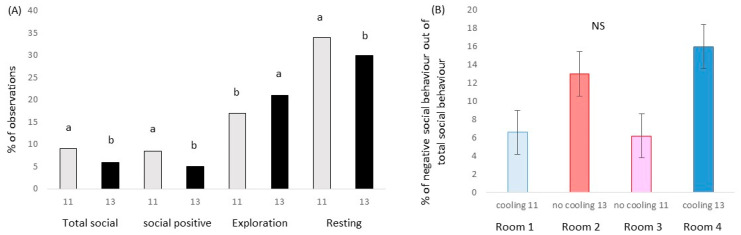
(**A**) Percentage of animals found performing social behavior (positive and/or negative; total social); just positive (social positive), exploratory behavior (exploration) or resting in relation to the number of animals in the pen (11 vs. 13). (**B**) Percentage of negative social behavior out of total social behavior in room with cooling system and low stocking density), room without a cooling system and high stocking density, room without a cooling system and low stocking density and room with a cooling system and high stocking density. Different letters mean significant differences at *p* < 0.05. NS means no significant differences at *p* > 0.05.

The experimental treatments also had an effect on pig behavior, as shown in Figure 4. A stocking density effect was found for positive social behavior (*p* = 0.006; χ^2^ = 7.5; DF = 1) and the social behavior as a sum of positive and negative (*p* = 0.018; χ^2^ = 5.6; DF = 1). Animals in higher densities showed fewer social behaviors when compared to those raised at lower densities. In addition, there was a trend of increased positive behaviors in males than in females (*p* = 0.096; χ^2^ = 2.8; DF = 1). Although negative social behavior was not affected by the treatments, the percentage of this behavior, in relation to all the social contacts, was double that in rooms with the lowest densities. In fact, a trend for cooling x density interaction (*p* = 0.087: χ^2^ = 2.9; DF = 1) was found for the percentage of negative social behavior out of the total social behavior. In the case of exploratory behavior, an effect of density was found (*p* = 0.026; χ^2^ = 5.0; DF = 1), with animals with higher densities showing this behavior more frequently than in animals in lower ones. Finally, the frequency of animals resting was affected by stocking density (*p* = 0.029; χ^2^ = 4.8; DF =1) and a cooling × density interaction (*p* = 0.009: χ^2^ = 6.8; DF = 1). In this case, animals were seen resting less frequently in pens with 13 than with 11 individuals, and, in fact, the maximum of animals seen lying down were the ones in the room without cooling and 11 animals per pen (37%), and the ones with less were also housed without cooling but with 13 individuals per pen (27%). No other significant differences were found, neither for cooling nor sex.

### 3.3. Blood and Hair Samples

**Figure 5:**
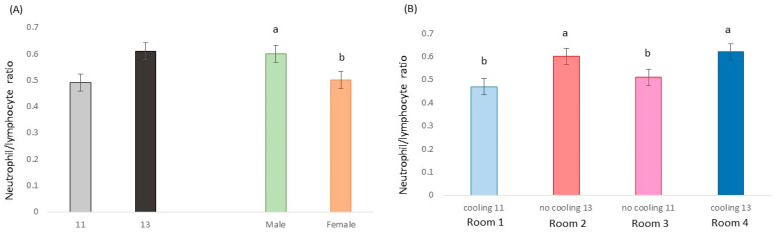
(**A**) The neutrophil/lymphocyte ratio in pens with 13 or 11 pigs and in pens containing only castrated males or females. (**B**) The neutrophil/lymphocyte ratio in animals in Room 1 (with cooling system and 11 animals per pen), Room 2 (no cooling and 13 individuals), Room 3 (no cooling and 11) and Room 4 (cooling and 13). Different letters mean significant differences at *p* < 0.05.

The neutrophil/lymphocyte ratio was influenced by the stocking density (*p* = 0.019; F = 5.6; DF = 1/174) and sex (*p* = 0.032; F = 4.7; DF = 1/174) but not by cooling (*p* = 0.772; F = 0.1; DF = 1/174). However, a cooling × density interaction was found (*p* = 0.047; F = 3.1; DF = 1/174), Figure 5.

**Figure 6:**
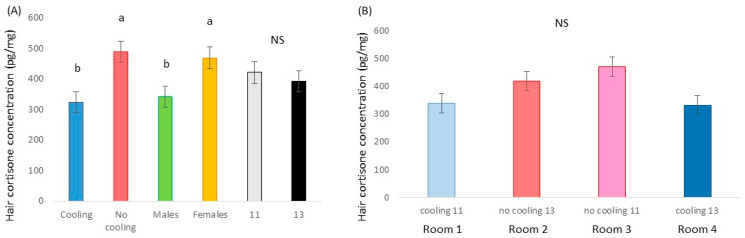
(**A**) Hair cortisone concentration in pigs housed in rooms with a cooling or without a cooling system, for males and females and when housed in low or high densities. (**B**) Cortisone concentration in hair in animals in Room 1 (with cooling system and 11 animals per pen), Room 2 (no cooling and 13 individuals), Room 3 (no cooling system and 11 animals per pen) and Room 4 (with cooling system and 13 animals per pen). Different letters mean significant differences at *p* < 0.05; NS means no significant differences.

Hair cortisone was affected by cooling (*p* < 0.001; F = 12.4; DF = 1/176), animals in pens without cooling having a higher concentration of cortisone than animals housed with cooling, and by sex (*p* = 0.009; F = 6.89; DF = 1/176), females having a higher concentration than males. In addition, a tendency for the stocking density*cooling interaction was found (*p* = 0.091; F = 2.90; DF = 1/176) (Figure 6).

## 4. Discussion

### 4.1. Thermal Conditions

As expected, the combination of temperature and humidity was lower (71) in rooms with a cooling system than in rooms without this evaporative device (75), confirming that pigs were subjected to the worst environmental conditions in the absence of this refreshing system [19]. In fact, taking into account the mean and maximum temperatures combined with the relative humidity, it can be concluded that animals with cooling were most of the time in a heat stress alert zone with some moments in the heat stress danger zone, while animals from the no cooling pens were most of the time in a heat stress danger zone with some periods in the heat stress emergency zone [20]. In fact, taking into account that thermoneutral temperatures for pigs range from 15 to 28 °C (EFSA [21]), in rooms without any refreshment device, these reached 32 °C.

### 4.2. Animal-Based Indicators

Pigs raised with both HS mitigation strategies (i.e., with cooling and at lower stocking densities) already weighed 2 kg more than the other treatments at the beginning of the study (15 d after mixing animals). During the trial, those pigs showed the highest ADG regardless of the initial BW. As expected, cooling systems succeeded at reducing the negative consequences of HS, but this effect was only significant when the pens had the lowest densities (Figure 2b). One of the factors explaining the differences in performance due to HS mitigation strategies is feed intake. Reducing feed intake is a key adaptive behavioral change in response to HS across species aimed at decreasing postprandial metabolic heat production [5,22]. In the future, other strategies already tested for thermal stress conditions, such as changes in the diet [23] and feeder space [24], could be tested in combination with the use of cooling systems and reduced stocking densities.

Another adaptive behavioral change of heat-stressed pigs raised in intensive conditions is to wallow in the manure to cool themselves off instead of in mud, as they would do if they were outdoors, to avoid contact with other pigs [4]. However, no difference in prevalence of dirtiness was found between pigs raised at the different stocking densities tested, and no clear effect of the cooling system was found. For instance, the room with cooling and 13 pigs per pen was not statistically different from the room without cooling and 11 pigs per pen. This does not agree with the results of Dalmau et al. [25], who concluded that the first reaction of pigs subjected to high temperatures was to lie on the manure, so animals were dirty before any change in physiological parameters could be observed. However, it is important to note that in the present study, this indicator was taken only once at the end of the growing period when the temperatures were not as high (Figure 1) as in previous weeks. In addition, Oliveira et al. [23] described a reduction in pig dirtiness at the end of the growing period (under identical temperatures as at the beginning), suggesting an adaptation period for the animals when high temperatures were maintained. Finally, another potential explanation, not being any of them mutually exclusive, is based on the fact that pigs raised at higher stocking densities dedicated less time to resting and more time to exploring, presumably because they had less available space in the pen to lie down, and this may have reduced the likelihood to soil themselves.

### 4.3. Blood and Hair Samples

The neutrophil/lymphocyte ratio and hair cortisone were assessed as indicators of mid-term and chronic HS, respectively. The neutrophil/lymphocyte ratio is a well-known indicator of stress; however, although it seemed to confirm that for pigs under high temperatures, it was better to stay in groups of 11 (0.80 m^2^ per pig) than of 13 animals (0.68 m^2^ per pig), it does not appear to be an indicator of HS. Previous research by Contreras-Jodar et al. [22] may confirm this hypothesis since HS, and the consequent reduction in feed intake, caused a decrease in hematopoiesis to spare the protein needed to create new cells and, thus, invest the protein to repair existing cells damaged by HS. Hence, an increase in neutrophil lineage was not expected. On the other hand, pigs raised with a cooling system during the growing–fattening period had less hair cortisone than those raised without a cooling system, indicating lower levels of cumulative stress. Therefore, both mitigation strategies induce a lower stress response in pigs reared in hot conditions, especially when both are combined.

### 4.4. Sex Effect

In the present study, castrated males were heavier (3.8 kg at the end of the study), dirtier and had a higher neutrophil/lymphocyte ratio than females. These three results could be linked between them by the fact that in a pen, the heavier pigs are the most susceptible to HS, resulting in worst neutrophil/lymphocyte ratio values and being dirtier because of wallowing in the manure. However, this does not explain the results found in hair cortisone, where females showed higher values than males. In this respect, further studies are needed to study the effect of sex on the cortisone concentrations in hair between male and female pigs.

## 5. Conclusions

Evaporative cooling and lowering stocking density reduced the negative effects of HS on the productive performance of growing–finishing pigs. The combination of both mitigation strategies resulted in the best productive performance. Each strategy showed improvements across different welfare indicators, such as hair cortisone and the neutrophil/lymphocyte ratio.

## Data Availability

Data is contained within the article.
